# The Multiple Sclerosis Prodrome: Evidence to Action

**DOI:** 10.3389/fneur.2021.761408

**Published:** 2022-01-31

**Authors:** Helen Tremlett, Kassandra L. Munger, Naila Makhani

**Affiliations:** ^1^Faculty of Medicine (Neurology), University of British Columbia, Vancouver, BC, Canada; ^2^Harvard T. H. Chan School of Public Health, Boston, MA, United States; ^3^Departments of Pediatrics and Neurology, Yale School of Medicine, New Haven, CT, United States

**Keywords:** multiple sclerosis, prodrome, preclinical, prevention, risk

## Abstract

A growing body of work points toward the existence of a clinically symptomatic prodromal phase in multiple sclerosis (MS) that might span 5–10 years or more. A prodrome is an early set of signs or symptoms predating the onset of classical disease, which in turn predates a definitive diagnosis. Evidence for a prodromal phase in MS could have major implications for prevention, earlier recognition and treatment, as well as an improved disease course or prognosis. This Perspective provides a succinct overview of the recent advances in our understanding of the MS prodrome and current key challenges. Many of the MS prodromal features characterized thus far are non-specific and are common in the general population; no single feature alone is sufficient to identify an individual with prodromal MS. Biomarkers may increase specificity and accuracy for detecting individuals in the MS prodromal phase, but are yet to be discovered or formally validated. Progress made in the elucidation of prodromal phases in other neurological and immune-mediated diseases suggests that these barriers can be overcome. Therefore, while knowledge of a prodromal phase in MS remains nascent, how best to move from the rapidly growing evidence to research-related action is critical. Immediate implications include refining the concept of the MS continuum to include a prodromal phase. This will help inform the true “at risk” period when considering exposures that might cause MS. Major long-term implications include the earlier recognition of MS, improved prognosis, through earlier disease management, and the future possibility of MS disease prevention.

## Introduction

A prodrome is an early set of signs or symptoms predating the onset of classical disease (1), which in turn predates a definitive diagnosis. Until recently, it was thought that multiple sclerosis (MS) did not have a prodromal period (1, 2), even though prodromal phases are well-recognized in other neurological and immune-mediated chronic conditions (3–6). While the prodrome remains a nascent field in MS, understanding the nature of the prodrome is critical in defining the etiologically relevant period when searching for risk factors for MS. Future applications may also include identification of individuals at risk of MS and enhanced opportunity for early management of disease. This Perspective Article summarizes the current state of knowledge of the MS prodrome, with a focus on the actionable evidence. Together with reflections on lessons learned from other chronic disease fields will help pave the path forwards to effect meaningful change in MS.

**Figure 1:**
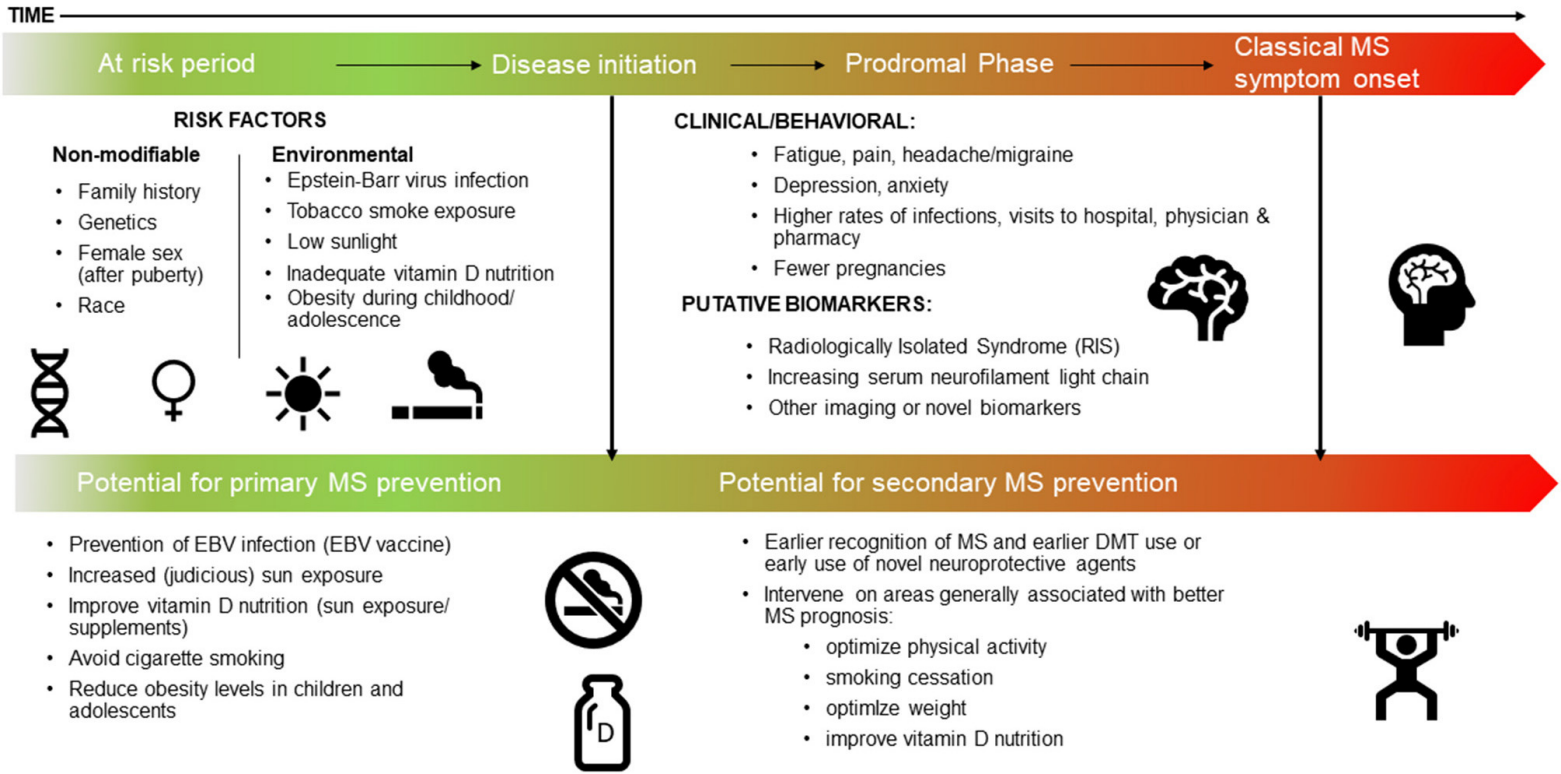
The MS continuum, a proposed timeline: the at risk period, the prodrome, and potential for prevention.

We focus here on the most recent literature, and studies not covered in detail in prior relevant articles (Box 1) (2, 7–10). We also include a brief overview of the most important or landmark findings to date, thus providing context to this rapidly emerging field. Each section concludes with a synopsis of the potential *actionable evidence*, thus providing an outline of how the field should harness knowledge of the MS prodrome to effect change, both now and in the future. Finally, we propose a refined timeline for MS, conceptualized as a continuum, which includes the prodromal phase (Figure 1).

Box 1 **Search strategy and selection criteria**

References for this article were identified by searching PubMed for journal articles published in English, with a focus on the last 5 years, using the following terms (and alternative spellings): *multiple sclerosis, prodrome, prodromal phase, pre-clinical, risk factor, RIS, CIS*. In addition, the reference lists of articles were reviewed along with the authors' own files and the most relevant articles were included within the article. The primary focus (selection criteria) were for peer-reviewed journal articles (original observational case-control, cohort or intervention studies, or other reviews of original work). Case reports and case series were excluded. Select older studies representing landmark advances were included, as necessary, in order to place current findings in context.

## The MS Prodrome: Key Findings

### The MS Prodrome: Clinical Aspects and Potential Duration

The last 5 years have seen the emergence of population-based studies which objectively measured signs and symptoms occurring before classical MS onset (8, 11–18). Importantly, the designs of these studies minimize the potential for both selection and recall bias. Collectively these studies suggest that an MS prodromal phase is detectable at least 5 years before MS symptom onset (or 10 years before a first MS diagnostic code), and possibly up to 20 years in persons who develop primary progressive (PP) MS (11–18). Studies in persons with radiologically isolated syndrome (RIS) suggest that the prodromal phase is of variable duration and may begin as early as 10–15 years before MS symptom onset (19, 20).

A myriad of signs and symptoms have been identified as more common during the years leading up to MS (defined by various studies as MS symptom onset or a first demyelinating code or a MS diagnostic code, Box 2), as compared to persons without MS, and range from cognitive deficits, to psychiatric morbidity, fatigue, sleep disorders, pain, fibromyalgia, bowel/bladder and dermatological issues (8, 11–18). In young men, aged 18 or 19 years old, entering the Norwegian military, lower cognitive scores were found in the 2 years before MS symptom onset, relative to those who did not develop MS (Δ = 0.80, 95% CI: 0.20–1.41, *p* = 0.0095, equivalent to a 6 IQ-point difference) (18). The mental health burden in the 5 years before a first demyelinating code or MS symptom onset, was measurable as ≈50% more visits to psychiatrics and ≈50% more mood disorder claims (based on physician-derived diagnostic ICD codes). Based on general practitioners records, depression may be more common up to 10 years before the first recorded MS or demyelinating diagnostic code (17). Intriguingly, the prodromal phase in children (first demyelinating diagnostic code <18 years of age) may have a negative impact on the mental health burden of their mothers; a possibility raised in one study (21). While the role of stress as a risk factor for MS onset remains unclear (22, 23), if a stressful event could trigger MS and also lead to mental-health related issues, this could provide an alternative explanation for findings. Finally, asymptomatic women at high (*n* = 27) vs. low (*n* = 20) risk of developing MS, based on a genes-environment score, exhibited poor vibration perception in their great toe [mean = 2.48 (SD: 0.60) vs. 1.83 (SD: 0.54), *p* = 0.008, age, height and test date adjusted] (24). Whether this represents a potential clinical sign of the MS prodrome is intriguing, but remains to be determined (24).

Box 2 **Identifying the MS prodromal phase**

*Definition of a prodrome*: an early set of signs or symptoms related to a disease, but predating the onset of classical symptoms, which in turn predates a definitive diagnosis.

*The challenge:* identifying the onset of classical disease can be difficult and differs across studies. For the purposes of this article, we have summarized the most common used below, and indicate what the timing (date) of each likely represents:

• *MS symptom onset*: typically recorded by a MS neurologist in a patient's medical record and is based on a careful medical history.

Represents the closest to actual classical onset of MS, based on current knowledge.

• First *demyelinating diagnostic code*: typically captured in health administrative data (from hospital or physician billing records) or in electronic medical records.

Represents the first formal medical recognition of a demyelinating event.

• First *MS diagnostic code* (e.g., International Classification of Diseases (ICD)-9/10 340 or G35, or Read codes): typically captured as for a first demyelinating diagnostic code.

Represents the first formal medical recognition of MS.

For the purposes of this article, ‘*classical MS onset'* is used to refer to either MS symptom onset or a first demyelinating code, as needed (e.g., to describe studies that used both to determine the end of the possible prodromal phase). MS symptom onset is arguably the closest possible to classical MS onset, thus enabling studies to avoid capturing the period between classical MS onset and diagnosis. This period, while of interest, should not be considered part of the prodromal phase.

### Patient Characteristics and the MS Prodrome

There is little research on whether the clinical presentation of the MS prodrome differs by age, sex, or the subsequent disease course (12, 16, 18). Current evidence suggests that pain is more evident in older adults while anemia is more pronounced in men, in the 5-years before a first demyelinating diagnostic code. The odds of pain increased from 1.76 (95% CI: 1.49–2.06) in those aged <30 years at their first event to 2.35 (95% CI: 2.13–2.60) in those ≥50 years (12), compared to matched controls without MS. The odds of anemia in men was higher [odds ratio (OR) 2.40; 95% CI: 1.68–4.29] than in women (OR: 1.23; 95% CI: 1.04–1.45), as compared to the general population. The sex-differences for anemia could simply reflect the higher prevalence of anemia among women, resulting in the lower relative estimate than men (12). Why anemia was more common for both men and women with MS during the prodrome is less clear. Findings could result from MS-related symptoms, such as fatigue, leading to an increase in detection of anemia among persons with MS (12). Intriguingly, recent work has suggested that red blood cells are active participants in the body's immune response (25), such that the inflammatory processes of MS could lead to a reduction in circulating red blood cells, leading to anemia.

Of the limited studies where disease course was examined (16, 18), those with either PP or relapsing-onset MS appeared to exhibit broadly similar prodromal features, with a notable exception for dermatological issues (16). In the 5 years before MS symptom onset, PP relative to relapsing-onset MS cases exhibited 47% lower rates of visits to dermatologists (rate ratio: 0.53; 95% CI: 0.30–0.96). Skin-related manifestations are recognized as relatively common in other immune-mediated diseases (26). Thus, whether these observations in MS indicate that early markers of inflammation differ by disease course, being lower in PP-onset MS cases is an intriguing possibility. Further, findings from the Norwegian military cohort suggest a much longer prodromal phase in PPMS; lower cognitive scores were measurable up to 20 years before PPMS symptom onset, relative to 2-years for the RRMS cases (21). For the PPMS cases, this was equivalent to 4.6-7 IQ-point difference compared to the control men who did not develop MS, *p* = 0.045 (21). While all these findings are interesting, confirmation in other, ideally larger populations is needed. Finally, no study to date has examined socio-demographic factors (e.g., race/ethnicity, socio-economic status, education or related health inequities), despite evidence that these are associated with MS outcomes after diagnosis (27, 28).

### Misdiagnosis and Missed Opportunity

Evidence of potential misdiagnoses and missed opportunities for earlier recognition of MS is also apparent across studies. For example, for individuals who developed PPMS, a higher rate of nervous system-related physician claims (ICD codes) in the 5 years before MS symptom onset was observed compared to relapsing-onset MS (rate ratio = 3.00; 95% CI: 1.06–8.49) (16). This may, in part, represent a delay in medical recognition which is not uncommon, particularly in PPMS (29, 30). Others have explored the issue of missed opportunities for earlier recognition by examining ambulatory care records in the years before a first MS diagnostic code (ICD 340) in a subgroup of patients with no record of a CIS and found that many physician visits in these patients before MS diagnosis were, in hindsight, likely a demyelinating event (31). These studies provide further evidence that earlier recognition of MS may be possible (31, 32).

#### Actionable Evidence

Together, these studies demonstrate that clinical features suggestive of an MS prodrome can be objectively measured at the population-level. Clearly, many of the MS prodromal features identified are also non-specific and common in the general population; no single feature alone will be sufficient to identify an individual with prodromal MS. Findings also suggest that an iterative approach is required; as earlier recognition and diagnosis of MS is achieved, then this could refine understanding of the MS prodrome. Thus, there is sufficient evidence to warrant further investment of resources and research funds in this area. The Table provides key examples. One low-cost, but valuable endeavor would be to re-evaluate previous studies for signs and symptoms suggestive of the MS prodrome.

## Putative Biomarkers of the MS Prodrome

Given the wide range of common and non-specific clinical symptoms observed at the population-level before the onset of MS symptoms, biomarkers for prodromal MS would be tremendously helpful. Such biomarkers could increase specificity and accuracy for identifying individuals in the MS prodrome.

### Neuroimaging and the Radiologically Isolated Syndrome

One potential biomarker is abnormal neuroimaging, such as in people with RIS. RIS is the clinical syndrome in which individuals underwent MRI scans of the brain for reasons other than suspected MS, resulting in an MRI finding suggestive of MS (i.e., this was an unexpected or incidental finding) (33). Formal criteria for RIS were proposed in 2009, which require that MRI findings meet the 2005 MRI criteria for dissemination in space (33). RIS differs from MS in that no classical MS symptoms are present. While some people with RIS are asymptomatic (e.g., they were participants in a research study), it can be inferred from the indications for obtaining MRIs that many have one or more non-specific symptoms, some of which potentially overlap with those of an MS prodrome. Such symptoms include mood disorders and, most commonly, headache (19, 20, 33). A substantial proportion of individuals with RIS (34% within 5 years and 51% within 10 years) subsequently developed a typical symptom of MS in sizeable multi-site studies (19, 20). While headache was not associated with an increased risk of subsequent clinical demyelination in one such study, the risk associated with other symptoms remains unknown (16). The precise relationship between RIS and an MS prodrome needs to be better understood, including whether they are distinct entities, overlapping entities, and/or part of a continuum (Figure 1). Given the possibility of overlap between potential symptoms of the MS prodrome (which commonly occur in the general population) and the non-specific symptoms reported in many people with RIS, RIS may emerge as being associated with prodromal MS. This possibility also provides rationale for exploring other neuroimaging biomarkers for the MS prodrome.

### Advanced Neuroimaging Techniques

Advanced neuroimaging techniques, when studied in the context of RIS, may also be useful for identifying biomarkers of the MS prodrome. For instance, regional (cerebellum and thalamus), and whole brain volumes were generally lower in individuals with RIS compared to controls (34–37). One study found that cortical volumes were similar in individuals with RIS (*n* = 19) and MS (*n* = 26), but were lower in these 45 individuals together as compared to 21 controls (38). In those with RIS, lower cortical volumes correlated with reduced performance on cognitive testing, suggesting an important functional association with a potential prodromal symptom. Other case-control studies have shown microstructural changes in brain white matter using diffusion tensor imaging and altered metabolic pathways in individuals with RIS using brain proton magnetic resonance spectroscopy suggesting their potential utility (39, 40).

Brain white matter lesions on MRI commonly occur for reasons other than demyelinating pathology. Therefore, there is a need for biomarkers specifically for the white matter lesions due to MS. For example, central veins occurred more frequently in white matter lesions (detected on MRI using FLAIR^*^ at 3T) in individuals with MS as compared to those with migraine in one study (41). Various definitions of the “central vein sign” also distinguished individuals with CIS and/or MS from those with other conditions (42, 43). It would be of value for future studies to determine whether central veins within MRI lesions are associated with increased risk for the subsequent development of clinical MS in people with RIS who also present with various symptoms, currently considered non-specific. Paramagnetic rims around lesions may also be a novel MRI biomarker of value during the MS prodrome (44).

### Serum, CSF and Other Opportunities for Biomarker Discovery

Given that the pathobiology of MS has presumably started before the prodromal phase, exploring biomarkers associated with neuronal injury and loss, such as neurofilament light chain (NfL), while not specific to MS, may be useful for the prodrome. In a nested case-control study of US military personnel, serum NfL levels were elevated in 30 individuals who subsequently developed MS as compared to 30 matched controls (median 16.7 vs. 15.2 pg/mL*, p* = 0.04) (45). Serum samples were obtained a median of 6 years before MS symptom onset in cases.

While less easily acquired than serum, CSF is often obtained in the diagnostic workup of individuals with suspected MS and it is therefore worthwhile to consider potential CSF biomarkers for the MS prodrome. In a study of 75 individuals with RIS, both unique CSF oligoclonal bands and elevated CSF NfL level were associated with the earlier development of CIS (hazard ratios 14.7, 95% CI: 1.8–120.2; *p* = 0.012 and 1.02, 95% CI: 1.00–1.04; *p* = 0.019, respectively) (46). Preliminary studies suggest that novel CSF analyses including single cell RNA sequencing may also hold promise. In one study, single cell analyses discriminated between the CSF immune profiles of twins discordant for MS (47). Other emerging evidence indicates that the gut microbiome may be altered in MS, suggesting another potential biomarker of the prodrome (48). Other potential molecular biomarkers include serum/CSF glial fibrillary acidic protein, and serum-based micro-ribonucleic acids (miRNAs) (49–51). Finally, abnormal visual evoked responses (52, 53) and optical coherence tomography (54) may be biomarkers associated with abnormalities in the visual pathways.

#### Actionable Evidence

Together these findings suggest that there may be measurable biomarkers for the MS prodrome including those in serum, CSF, and on MRI that warrant further study (see Table 1). Exploration of biomarkers for the MS prodrome will likely result in an improved understanding of the pathology of MS itself as many of these biomarkers reflect underlying pathophysiological mechanisms.

**Table 1:** Actionable evidence and the MS prodrome: from general to specific examples.

Clinical aspects of the MS prodrome	
Evidence	Clinical features of the MS prodrome can be objectively measured 5–10 years before classical MS onset (8, 11–18).
*Action*	*Invest further resources to support research of the clinical features of the MS prodrome*
	*Systematically re-evaluate previous studies for signs and symptoms suggestive of the MS prodrome*
	*Determine what the potential duration of the MS prodrome is, and whether, and how this differs across populations and within specific patient groups*
	*Refine the proposed timeline for MS, conceptualized as a continuum, to include the prodromal phase, see Figure (i.e., the at risk phase precedes disease initiation, which is then followed by the prodromal phase, classical MS symptom onset and diagnosis)*
Evidence	At the population-level, specific clinical features (often derived from health administrative data or medical records) are more common for MS cases at least 5 years before MS symptom onset, and possibly up to 20 years in PP MS, relative to population controls (12–14, 16, 18).
*Action*	*Greater granularity is required to capture subtle signs and symptoms of the MS prodrome that do not necessarily prompt medical attention*
Evidence	Features of the MS prodrome may differ by sex, age and disease course (12, 16, 18)
*Action*	*Investigate how patient characteristics may influence presentation of the MS prodrome (e.g., age, sex, socio-economic status, race/ethnicity/culture, health inequity) and how features of the MS prodrome may differ by subsequent disease course*
Evidence	Some clinical features captured before MS symptom onset (16), or a first MS diagnostic code (31), are suggestive of misdiagnoses and missed opportunity for prompt recognition and earlier appropriate MS diagnosis
*Action*	*Need to better understand why and when these are occurring and if amenable to change, resulting in improved outcomes for people with MS*
	*Iterative approach required; earlier recognition and diagnoses of MS will refine understanding of the MS prodrome*
Biomarkers of the MS prodrome	
Evidence	Many individuals with radiologically isolated syndrome have symptoms that overlap with prodromal MS and subsequently develop classical MS symptom onset (19, 20, 33)
*Action*	*Determine the relationship between radiologically isolated syndrome and the MS prodrome*
	*Other neuroimaging biomarkers for the MS prodrome warrant study*
Evidence	Serum neurofilament light is elevated up to 6 years before MS symptom onset (45)
*Action*	*Establish whether other biomarkers in the CSF and serum as well as novel biomarkers, such as the composition of the gut microbiome, may be measurable prior to, and are associated with, subsequent classical MS symptom onset*
Risk factor (trigger) or prodromal feature?	
Evidence	Many studies focus on exposures during the few years before reported MS symptoms onset or MS diagnosis
*Action*	*Re-evaluate the MS environmental risk factor literature to determine whether associations may be due to exposure being measured in the presumed prodromal phase rather than the true “at risk” period*
	*Future study designs of environmental risk factors need to factor in the timing of a possible prodromal phase*
Prodromal phases in other diseases and implications for MS	
Evidence	Criteria exist to identify other prodromal diseases; e.g., validated research criteria to identify likely prodromal Parkinson's disease (55)
*Action*	*Examine the feasibility (including key gaps in knowledge), and the acceptability of developing research criteria for prodromal MS*
	*Develop research criteria to identify the probability of a person having prodromal MS*

## Risk Factor (Trigger) or Prodromal Feature?

The duration of the MS prodrome can be defined as the time between the initiation of MS pathology and the appearance of the classical clinical demyelinating events that eventually lead to an MS diagnosis (Figure 1). Knowledge of this period is critical to identify true causal risk factors for MS. Many environmental exposures assessed after the MS disease process begins may not be an accurate representation of the pre-pathological onset exposure. For example, during the prodromal phase, general feelings of unwellness may lead to changes in diet or physical activity, and any associations observed are more likely to be due to “reverse causation” and not a true causal risk factor.

There are currently four environmental risk factors for MS that evidence suggests may have a causal role in MS development: infection with Epstein-Barr virus (EBV), low sunlight exposure/low serum vitamin D levels, obesity in early life, and cigarette smoking (56). One necessary determinate of causality is temporality—i.e., the exposure must occur before the initiation of the disease process. While there is evidence supporting temporality for each of these factors based on childhood/adolescent exposure being associated with future MS risk, a closer look at the lower risk of MS with higher serum 25-hydroxyvitamin D (25(OH)D) levels is illustrative of the complexity of whether a risk factor is a trigger or prodromal feature. There have been four prospective studies of serum 25(OH)D measured in samples collected on average 5, (57) 8, (58) and 9 (59, 60) years before MS symptom onset with an overall range of less than one to up to 32 years and all found an inverse association between higher 25(OH)D levels and risk of MS onset. That the average time of sample collection before MS onset in these studies falls within 10 years before MS symptom onset, and the possibility that 25(OH)D levels decline during a prodromal phase (e.g., if an individual begins sun avoidance behaviors due to not feeling well), reverse causation cannot be ruled out as a possible explanation on the basis of these results alone. Results of two studies of 25(OH)D levels during pregnancy or at birth and future risk of MS in the offspring found that deficient serum vitamin D levels in mothers or in dried blood spots from neonates were associated with an increased risk of MS onset in the child (61, 62), and case-control studies of sun exposure have consistently found an inverse association between higher sun exposure in childhood/adolescence and lower MS risk (56, 63–65). Additionally, Mendelian randomization studies have found that genetically lower 25(OH)D is associated with an increased risk of MS in adults and children (66–68). Together, these studies suggest that exposure to low vitamin D levels may pre-date the onset of the prodromal phase and be a true risk factor for MS. EBV infection is also a risk factor for MS and the evidence for infection occurring prior to the onset of MS, and the prodromal phase, is strong (56). Individuals who are EBV seronegative have a near zero risk of having MS, and a prospective study among EBV seronegative young adults found the risk of MS increased only after infection with EBV (69). There was no increase in risk of MS with infection of cytomegalovirus (as measured serologically) over the same time period (69), suggesting the association is EBV specific rather than a general increased risk of infections.

Studies of other risk factors that have been measured within the presumed prodromal phase, i.e., within 5–10 years of MS symptom onset, include migraines, lower levels of physical activity, diet quality, pregnancy and oral contraceptive use (70–73). Pregnancy, for example, has been associated with a decreased MS risk, while oral contraceptive use associated with an increased risk in some studies (73), but studies of the MS prodromal phase suggest that women who develop MS may choose birth control or delay pregnancy simply because they are experiencing signs and symptoms of the prodromal phase (14). Similarly with diet quality before MS symptom onset, no association with MS risk was found, but if individuals make dietary improvements in response to prodromal sign and symptoms, reverse causation may be one explanation (70). Defining the true time of MS onset and studying exposures before that time is critical in teasing apart risk factors from prodromal features.

### Actionable Evidence

Given the evidence that prodromal MS may precede MS symptom onset by 5 or more years, a review and re-evaluation of the MS environmental risk factor literature should be conducted to determine whether any associations (null or otherwise) may be explained by the exposure being measured in the presumed prodromal phase rather than before. Further, future study designs of environmental risk factors of MS need to factor in the time of a possible prodromal phase, assessing exposure at multiple time points prior—perhaps up to 10 years or more–to MS symptom onset, though this is not without challenges.

## Future Perspectives

Evidence for a prodromal phase of MS has major implications for prevention, earlier recognition and diagnosis of MS, as well as improved disease prognosis. Immediate implications include refining the conception of a timeline for MS that includes a prodromal phase as part of the MS continuum (Figure 1). This will help inform the true “at risk” period when considering risk factors that might trigger or cause disease initiation and onset of MS. As our understanding of the possible duration of the MS prodrome is refined, this will provide further clarity and advance capacity to potentially prevent MS though interventions implemented before disease initiation and the onset of clinical MS (that is during a “true” risk factor phase). Of note, it is feasible that there will be overlap between risk factors for MS and features of the MS prodrome. For example, it is reasonable to expect serum vitamin D to be low during the prodromal phase as people change behavior in response to increasing health concerns, and consequently spend less time outdoors. However, low serum vitamin D levels earlier in life may also increase the risk of developing MS, in certain populations.

While longer term implications of the MS prodrome include the potential for earlier recognition or diagnosis of MS, much more work is needed before this could be applied in clinical practice. Also, while studies to date have provided a “proof-of-principle” that an MS prodrome exists, many of the individual features identified are not specific to MS and are common in the general population. However, a tangible future goal, which could facilitate improving outcomes in MS, could be the development of *research* criteria for prodromal MS. A probability score, estimating the likelihood of an individual being in the prodromal phase of MS is envisaged. Building on current knowledge, including prior MS genetic risk scores (74–76), this prodromal probability score could be based on an optimal combination of prodromal clinical features (e.g., depression, anxiety, pain, dermatological issues or other combinations of features) with risk markers (e.g., age, sex, family history/genetics) and biomarkers (e.g., serum NfL, imaging markers [such as those observed in people with RIS], serum vitamin D). This approach is similar to the research criteria developed to identify prodromal Parkinson's disease (55, 77) and to those being tested/developed in other neurodegenerative and autoimmune diseases including dementia with Lewy bodies (78), Type 1 diabetes (79), and rheumatoid arthritis (4). Such research criteria could facilitate identification of high-risk individuals, defined using an acceptable threshold, e.g., 80 or 90% probability of having prodromal MS. This information is envisaged for research purposes only (not clinical practice). For example, these individuals could be offered enrollment in clinical trials of future neuroprotective drugs or other interventions (80). This would complement the ongoing clinical trials in people with RIS in which disease-modifying drugs approved to treat MS are being tested for their ability to prevent or to delay classical MS symptom onset (e.g., NCT02739542, NCT03122652). Creation of validated research criteria for prodromal MS will require further research investment to provide greater granularity of the most relevant prodromal features (Table 1) and will ultimately require contributions from a broad range of international stakeholders, including multi-disciplinary researchers, clinician-scientists and the MS community.

## Data Availability Statement

The original contributions presented in the study are included in the article, further inquiries can be directed to the corresponding author/s.

## Author Contributions

HT, KM, and NM drafted the original manuscript, reviewed and edited the text, Tables, Boxes, and Figure. All authors contributed to the article and approved the submitted version.

## Funding

This work was supported, in part, by the National Institute of Neurological Disorders and Stroke of the National Institutes of Health under award number K23NS101099.

## Conflict of Interest

NM was funded by NIH/NINDS (Grant No. K23NS101099) and the Charles H. Hood Foundation. KM was funded by NIH/NINDS and the US Department of Defense. She has received travel expenses to participate in conferences from the National MS Society (2019, 2020) and ECTRIMS/ACTRIMS (2018, 2019). She was a paid consultant for serving on a scientific advisory committee (Biogen, 2020). HT was the Canada Research Chair for Neuroepidemiology and Multiple Sclerosis. Current research support received from the National Multiple Sclerosis Society, the Canadian Institutes of Health Research, the Multiple Sclerosis Society of Canada and the Multiple Sclerosis Scientific Research Foundation. In addition, in the last 5 years, has received research support from the UK MS Trust; travel expenses to present at CME conferences from the Consortium of MS Centres (2018), the National MS Society (2016, 2018), ECTRIMS/ACTRIMS (2015–2020), American Academy of Neurology (2015, 2016, 2019). Speaker honoraria are either declined or donated to an MS charity or to an unrestricted grant for use by HT's research group. Unrelated to this study NM receives funding from the Charles H. Hood Foundation.

## Publisher's Note

All claims expressed in this article are solely those of the authors and do not necessarily represent those of their affiliated organizations, or those of the publisher, the editors and the reviewers. Any product that may be evaluated in this article, or claim that may be made by its manufacturer, is not guaranteed or endorsed by the publisher.
